# Recent advances in *in vivo* applications of intein-mediated protein splicing

**DOI:** 10.1186/1759-8753-5-5

**Published:** 2014-02-04

**Authors:** Natalya I Topilina, Kenneth V Mills

**Affiliations:** 1 Department of Biological Sciences, University at Albany, State University of New York, 1400 Washington Avenue, Albany, NY 12222, USA; 2 Department of Chemistry, College of the Holy Cross, 1 College Street, Worcester, MA 01610, USA

**Keywords:** Intein, Protein splicing, Post-translational modification, Protein purification, *Trans*-splicing, Conditional protein splicing, *Trans*-genes, Biosensor

## Abstract

Intein-mediated protein splicing has become an essential tool in modern biotechnology. Fundamental progress in the structure and catalytic strategies of *cis-* and *trans-*splicing inteins has led to the development of modified inteins that promote efficient protein purification, ligation, modification and cyclization. Recent work has extended these *in vitro* applications to the cell or to whole organisms. We review recent advances in intein-mediated protein expression and modification, post-translational processing and labeling, protein regulation by conditional protein splicing, biosensors, and expression of *trans*-genes.

## Introduction

**Figure 1:**
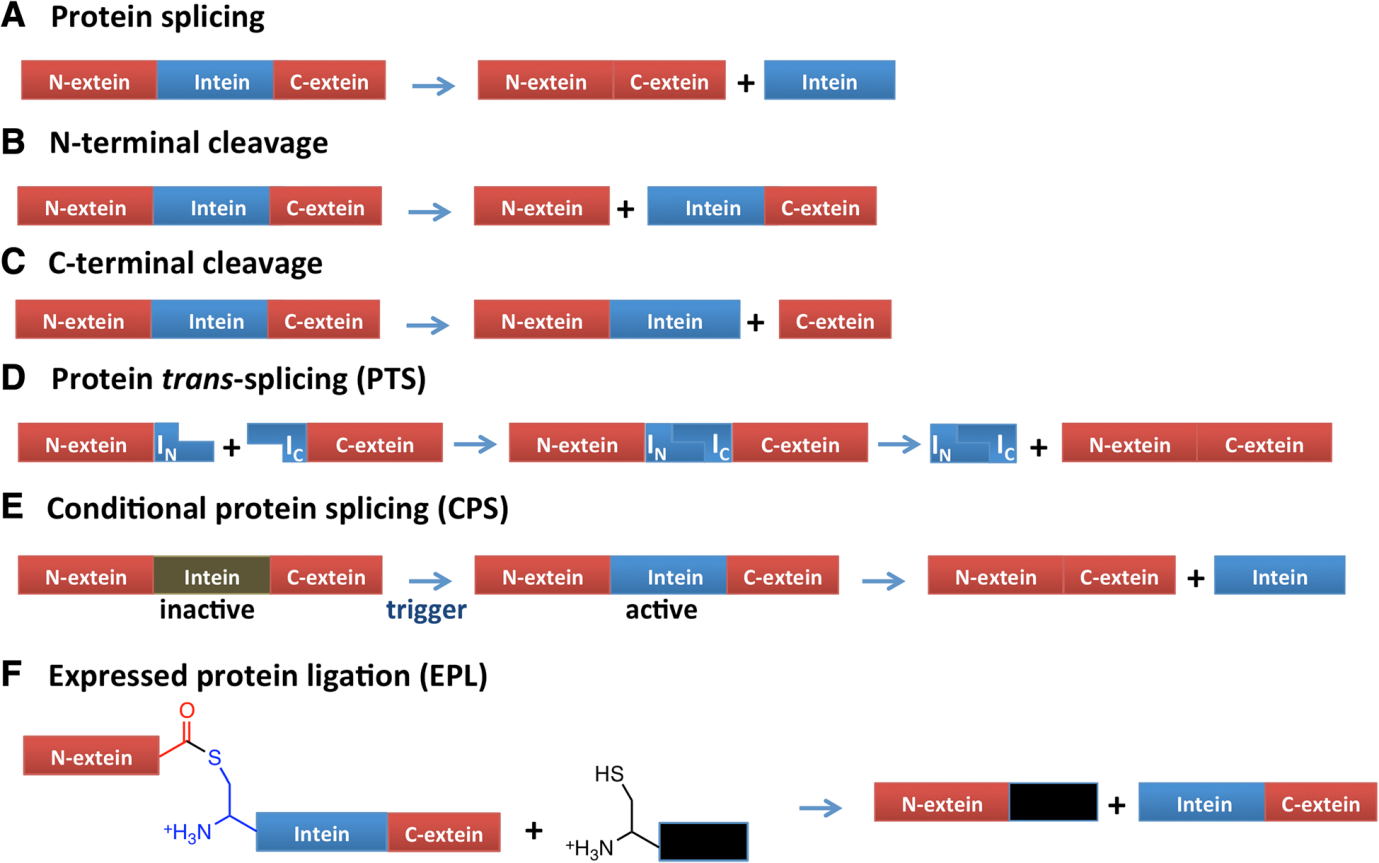
**Schematics of protein splicing, side reactions, *trans*-splicing, conditional protein splicing and expressed protein ligation. A.** Protein splicing. **B.** N-terminal cleavage side reaction. **C.** C–terminal cleavage side reaction. **D.** Protein *trans-*splicing (PTS). **E.** Conditional protein splicing (CPS). The brown color of the intein box in E indicates an inactive intein, and the blue color is active. **F.** Expressed protein ligation (EPL). The black box can be a protein with an N-terminal Cys, either a synthetic peptide or a protein with an N-terminal Cys revealed by proteolysis or intein-mediated cleavage.

Protein splicing is a post-translational process by which an intervening polypeptide, called an intein, catalyzes its own excision from the flanking polypeptides, or exteins, as well as ligation of the exteins (Figure 1A).

Many inteins are interrupted by homing endonuclease domains similar to those found in mobile introns. However, the homing endonuclease domain can be deleted from the intein without complete loss of splicing activity and is absent in a class of inteins called mini-inteins [1].

**Figure 2:**
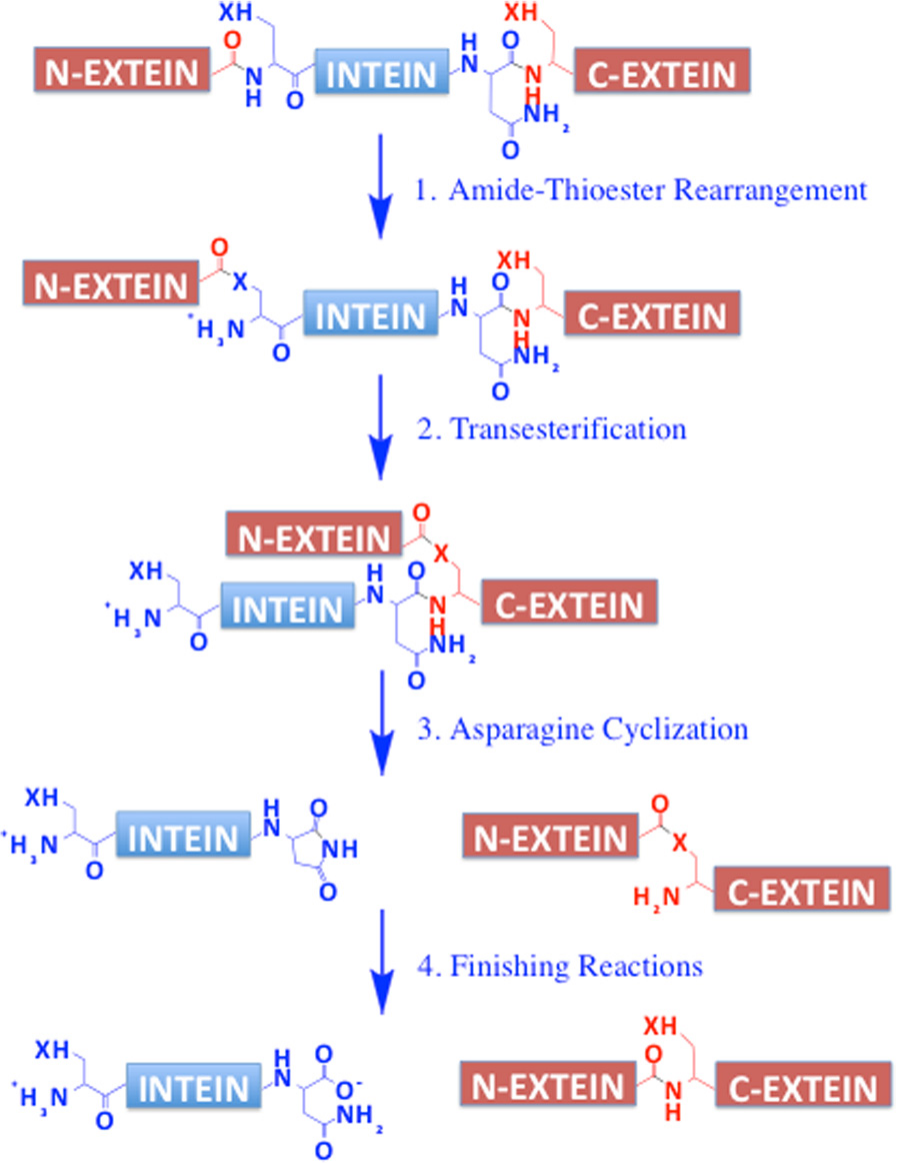
**Mechanism of protein splicing.** X = S or O, such that residue 1 or the intein may be Cys or Ser, and residue C + 1 of the C-extein may be Cys, Ser or Thr. The finishing reaction shows the production of C-terminal Asn, but may also produce iso-Asn.

The mechanism of splicing for canonical inteins is a four-step process (Figure 2) [1,2]. First, the peptide bond linking the N-extein and intein is converted to a thioester or ester via nucleophilic attack by the N-terminal Cys or Ser of the intein (Step 1). Second, the N-extein is transferred from the side chain of the first intein residue to the side chain of the first C–extein residue (Cys, Ser or Thr) by transesterification, resulting in a branched ester intermediate (Step 2). Third, the branched ester is resolved by Asn cyclization coupled to peptide bond cleavage (Step 3). This leaves the ligated exteins separated from the intein and linked by an ester bond, while the intein has a C-terminal aminosuccinimide. Finally, the ester bond connecting the ligated exteins is rapidly converted to the amide bond, and the C-terminal aminosuccinimide of the intein may be hydrolyzed (Step 4).

Two side reactions can occur if the splicing process is disrupted (Figure 1 B and C). The ester or thioester formed in Steps 1 and/or 2 may be cleaved by hydrolysis or thiolysis, uncoupled from Asn cyclization. This process is called N-terminal cleavage, and results in cleavage of the N-extein from the precursor (Figure 1B). Alternatively, Asn cyclization may occur uncoupled from Steps 1 and 2, freeing the C-extein in a process called C-terminal cleavage (Figure 1C).

Some inteins are expressed as two separate fragments, either natively or by protein engineering, and facilitate protein splicing in *trans* (Figure 1D). In protein *trans*-splicing, or PTS, reassociation of the intein fragments is required prior to splicing.

Both *cis-* and *trans-*splicing inteins have been engineered to undergo conditional protein splicing, or CPS (Figure 1E). CPS requires the addition of a trigger to initiate splicing of a precursor fusion protein. Such triggers include light, changes in pH or temperature, change in redox state, or the addition of a small molecule [2-6]. For CPS of *trans-*splicing inteins, split dimerization domains have been fused to the intein fragments to make reassociation conditional on addition of a small molecule or on the affinity of the domains [2-6].

Intein-based methods have been developed to facilitate purification and post-translational modifications of recombinant proteins. Expressed protein ligation (EPL) and protein *trans*-splicing (PTS) [7-9] can produce proteins with site-specific incorporation of a diverse set of chemical modifications [6,7,10-13]. We introduce these methods here and will describe more recent applications below.

EPL is a method to modify the C terminus of a recombinant protein [14,15] (Figure 1F). The protein is fused at its C terminus to an intein, which promotes formation of a thioester between the protein and intein. The protein is then transferred to the side chain of a synthetic peptide with an N-terminal Cys (or a protein with N-terminal Cys revealed by proteolysis or intein-mediated cleavage). The peptide may contain non-native amino acids or other chemical probes that can be incorporated by solid phase peptide synthesis. EPL is similar to native chemical ligation [16], which facilitates the ligation of a small synthetic peptide with a C-terminal α-thioester to a peptide with an N-terminal Cys. In most EPL strategies, the ligated segments have no natural affinity to each other and there is an entropic barrier to ligation. However, this entropic barrier can be overcome if the segments to be ligated have affinity for one another [17].

In PTS, natively or artificially split inteins ligate the exteins via a peptide bond [18,19]. The production of semi-synthetic proteins via PTS takes advantage of the affinity between the intein fragments. However, PTS relies on efficient splicing rather than on intein side reactions. Thereby it has additional challenges in that efficient splicing may depend more heavily on the presence of short native exteins and on where the target protein is split. As a result, one may need to incorporate short native extein sequence into the final ligation product or substantially optimize the split site.

Intein biotechnology applications have been extensively and expertly reviewed in the recent literature [2-6]. We aim to describe in detail the most recent advances in this area, including protein expression and modification, post-translational processing and labeling, protein regulation by conditional protein splicing, biosensors, and the expression of *trans*-genes.

## Review

### Protein expression and modification

Intein-based methods can be used to modify the sequence or structure of recombinant proteins, including protein cyclization or polymerization, expression of proteins with native N-terminal residues, and site-specific proteolysis. Inteins can facilitate the expression of toxic proteins and large proteins from within the same reading frame, can allow for post-translational generation of small peptides, and can serve as selectable genetic markers.

#### Cyclization

**Figure 3:**
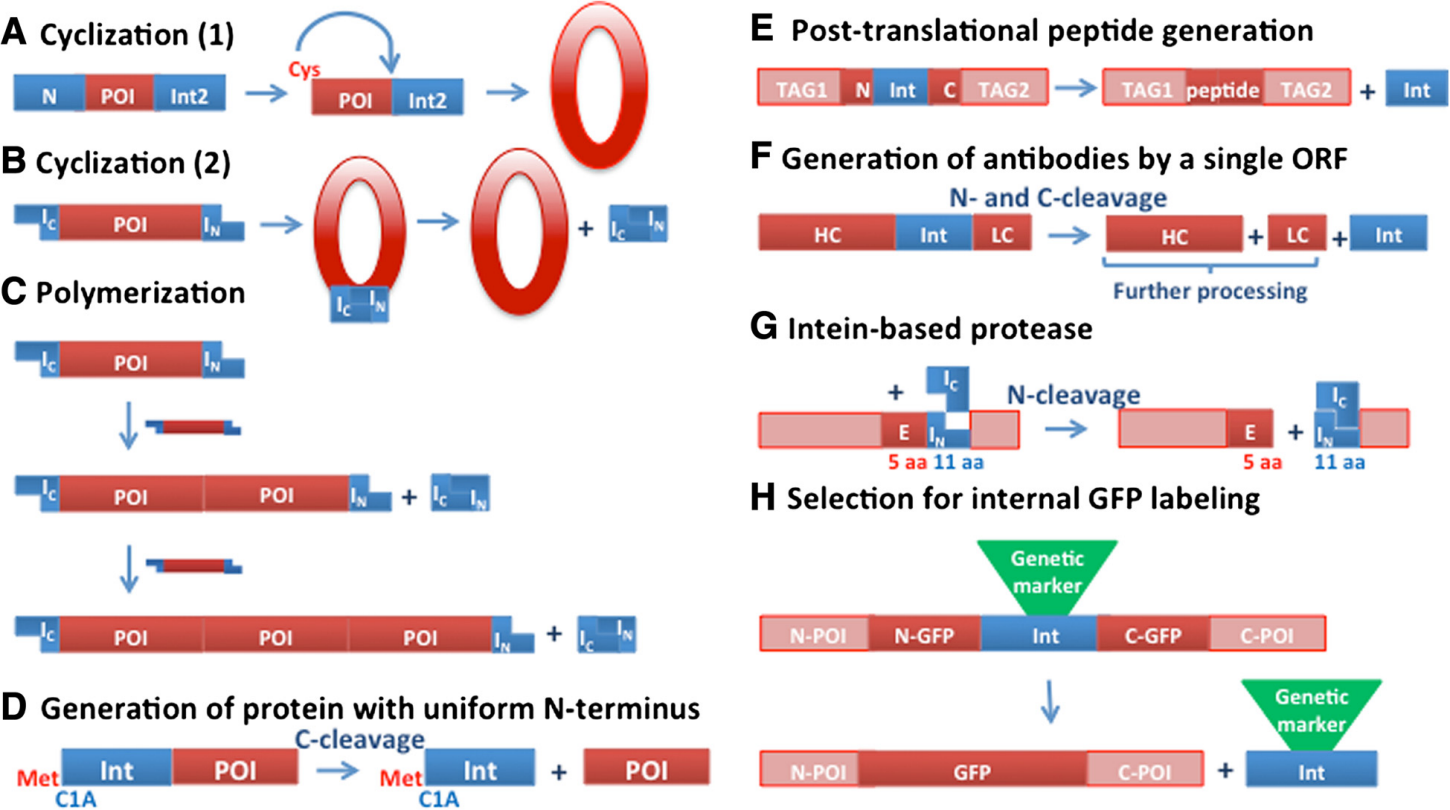
**Schematic representations of intein-mediated post-translational processing.** In all panels, POI indicates protein of interest. **A and B.** Intein-mediated cyclization. In A, 'N’ indicates either an intein that generates an N-terminal Cys on the POI by C-terminal cleavage or a protein or peptide removed by proteolysis to reveal an N-terminal Cys. **C.** Intein-mediated protein cyclization. **D.** Generation of a protein with a uniform N-terminal residue. **E.** Post-translational peptide generation. **F.** Generation of antibodies by a single open reading frame (ORF). HC indicates heavy chain and LC indicates light chain. **G.** Use of an intein as a highly specific protease. **H.** Selection for internal GFP labeling.

Protein and peptide cyclization can be facilitated by inteins by two methods (reviewed in refs [3,4,20,21].) In the first method (Figure 3A), cyclization can be achieved by having the protein of interest (POI) fused to different inteins at its N and C termini [22,23]. C-terminal cleavage at the N-intein/target junction results in an N-terminal Cys residue. Alternatively, an N-terminal Cys residue may be generated by proteolysis. This Cys reacts with an activated thioester generated at the target/C-intein junction via EPL to produce cyclized protein. In the second method (Figure 3B), split inteins are used to produce cyclized peptides or proteins. The target protein or peptide is expressed as a fusion between C- and N-split intein fragments [24,25]. The inversion of the placement of the N- and C-intein fragments in the precursor (I_C_-protein-I_N_) ensures that PTS results in ligation of the internal polypeptide fragment. The cyclization of the target proteins results in enhanced stability and bioactivity [26-31]. One of the most exciting applications of intein-mediated cyclization is the *in vivo* generation of large libraries of genetically-encoded cyclic peptides for high throughput screens [3]. In addition to cyclization, intein splicing from the I_C_-protein-I_N_ precursor may result in polymerization of the target protein [32] (Figure 3C).

#### Proteins with uniform N-terminal residues

Inteins have been used to express proteins in *E. coli* with uniform N-terminal residues [33,34]. For instance, proteins expressed in their host organism that have signal sequences often have that signal sequence cleaved by an aminopeptidase, resulting in a protein with an N-terminal residue other that Met. When these proteins are over-expressed in *E. coli* without their native signal sequences, they can be subject to unwanted N-terminal processing by aminopeptidases. To avoid this cleavage and have their native N-terminal residue, a target protein was fused at its N terminus to the *Ssp* DnaB mini-intein, and expressed in an aminopeptidase-deficient strain (Figure 3D). Upon C-terminal cleavage of the intein, the target protein with the desired N-terminal amino acid was generated [33,34].

#### Expression of peptides, toxic proteins, and proteins from a single reading frame

Intein technology can be used to express challenging targets: small peptides can be expressed as part of properly folded proteins with affinity domains, toxic proteins can be expressed in an inactivated format, and proteins that function in an essential stoichiometry can be expressed from a single open reading frame.

Intein catalysis can facilitate post-translational generation of peptides by protein splicing [35] (Figure 3E). Intein-mediated intracellular peptide production was used to distinguish between the behavior of peptides generated by cleavage of disordered, defective ribosomal products and those generated from well-folded proteins. For example, it was thought that major histocompatibility complex (MHC) class I peptides were mostly derived from cleavage of misfolded protein fragments. However, peptides produced via splicing of either the *Mtu* RecA or *Pch* PRP8 mini-inteins also generated MHC class 1 epitopes. Because the intein precursor protein must be stably folded to facilitate splicing, this suggests that MHC peptides can be produced from stable, well-folded proteins.

Inteins can facilitate the overexpression of toxic proteins. For instance, the *Sce* VMA intein has been used to create building blocks for the semi-synthesis by EPL of active cytotoxic enzymes from inactive fragments, including bovine pancreatic RNase A and a restriction endonuclease from *Haemophilus parainfluenzae* (*Hpa*I) [14]. Another approach is to produce non-toxic protein precursor by inserting an intein in the toxic protein, with activity of the target protein dependent on CPS of the intein. For example, I-TevI endonuclease was expressed by inserting a modified *Mtu* RecA intein that is active only under specific pH conditions [36,37].

Expression of antibodies using a single open reading frame was achieved by fusing the genes for antibody heavy and light chains with an intein [38] (Figure 3F). This fusion protein was successfully expressed and processed in mammalian cells, with intein-directed N- and C- terminal cleavage reactions resulting in antibodies with the correct sequences for both heavy and light chains.

#### Intein proteases

Split inteins can be used to facilitate *in vivo,* site-specific protein cleavage [39] (Figure 3G). The 11-residue N-terminal fragment of the *Ssp* DnaB S1 split intein was inserted between two target sequences and used as a cleavage site that is recognized by the C-terminal intein fragment. This C-terminal fragment is called an intein-derived protease (IP), because on co-expression of IP and complementation with the N-extein fragment, the target protein is cleaved via N-terminal cleavage of the reconstituted split intein. Site-specific protein cleavage by the IP was demonstrated in bacterial and eukaryotic cells. In contrast to the relatively low substrate specificity of other commonly used proteases, this intein-derived protease has very limited unintended proteolysis of endogenous proteins resulting in minimal cellular toxicity. The authors suggested utilization of the IP as a molecular tool to provide control of protein cleavage inside living cells.

#### Inteins as genetic markers

Inteins can facilitate *in vivo* gene modification by serving as genetic markers [40] (Figure 3H). Muller and coworkers interrupted the *Pch* PRP8 intein with selectable markers, including aminoglycoside phosphotransferase and imidazoleglycerol-phosphate dehydratase. The interrupted inteins are able to splice, and could serve as selectable markers for expression of the spliced extein, GFP. This split GFP (or GFP:int) construct was used for a single step internal labeling of calmodulin with GFP in yeast.

### Intein-mediated protein processing and labeling

EPL and PTS have been successfully used to produce diverse N- and C-terminal modifications of target proteins that are expertly reviewed in the recent literature [7,8,10,12], including protein phosphorylation, lipidation, glycosylation, biotinylation, ubiquitination, and segmental isotope labeling. Here we focus on *in vivo* protein modifications, including protein semi-synthesis on cell surfaces, segmental isotope labeling inside cells, and selective protein labeling inside of living cells.

#### Protein semi-synthesis on a cell surface

**Figure 4:**
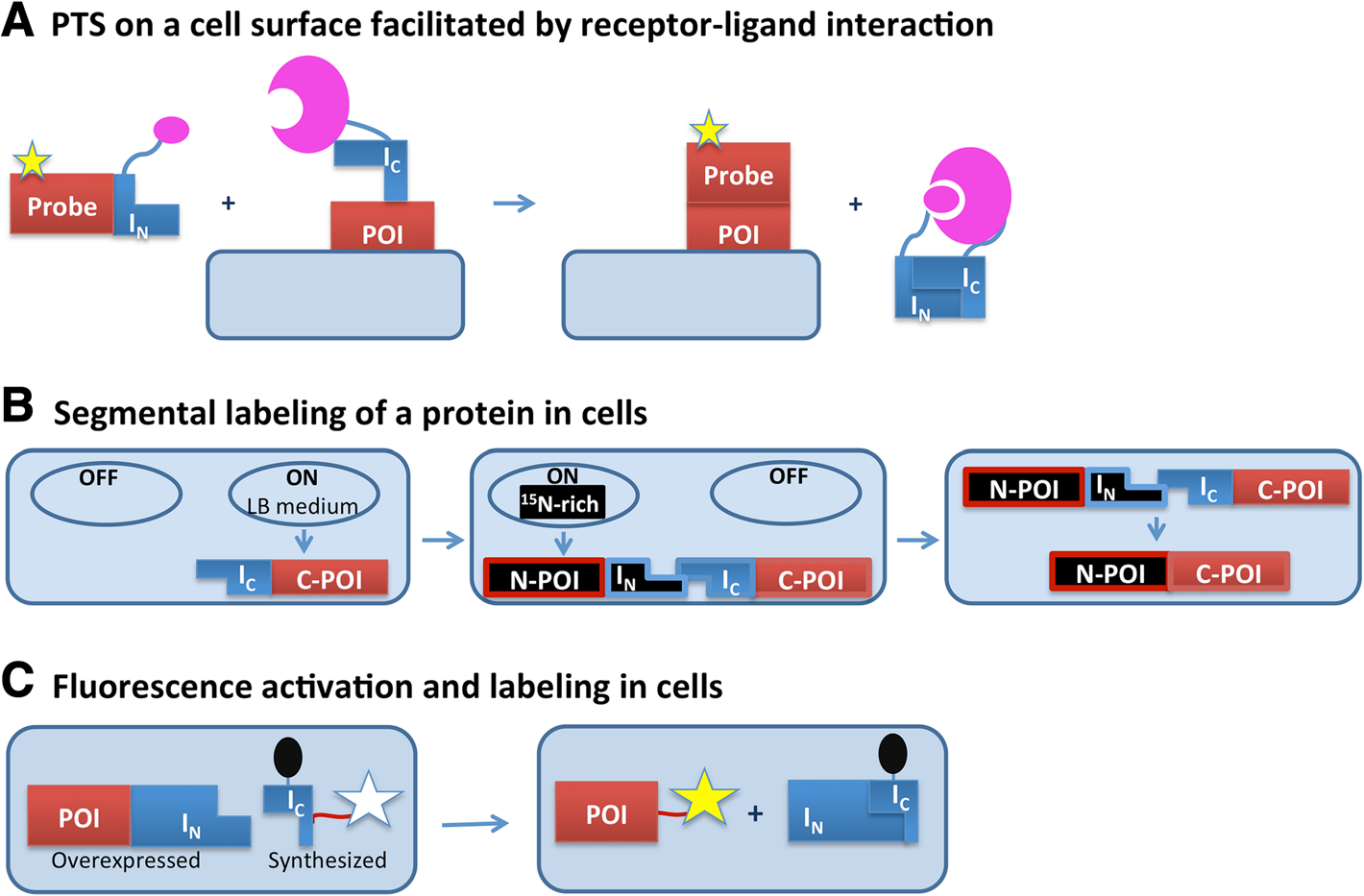
**Schematic representations of intein-mediated protein labeling.** POI is protein of interest. In **(A)**, the complementary pink shapes represent the ligand and its receptor. In **(B)**, 'off’ and 'on’ indicate conditional expression states from a plasmid, with the proteins in black ^15^ N labeled and those in blue or red are not labeled. In **(C)**, the conversion of the star from clear to yellow indicates induction of fluorescence.

PTS has been used for protein semi-synthesis on a cell surface (Figure 4A). The C terminus of the human transferrin receptor was labeled with a fluorescent group (5-carboxy-fluorescein) on the surface of Chinese hamster ovary (CHO) cells using the *Ssp* GyrB split intein [41]. Likewise, the N terminus of the monomeric red fluorescent protein (mRFP) was modified with a biotin tag via PTS on the surface of CHO cells [42]. PTS can be employed for ligation of an endogenous polypeptide to a membrane protein on mammalian cells [43]. Mootz and coworkers attached enhanced GFP (eGFP) to transmembrane- and GPI-anchored proteins via a PTS reaction between the *Npu* DnaE Int_C_ fragment fused with the membrane-localized protein and *E. coli* overexpressed eGFP-Int_N_ fusion.

To overcome the low binding affinity between split intein pairs that have a short N-terminal fragment, an auxiliary receptor-ligand interaction was integrated, as outlined in Figure 4A [42].

#### In-cell protein labeling

PTS can facilitate segmental isotopic labeling *in* vivo, as well as the *in vivo* addition of chemical probes to specific target proteins. Cell-based PTS can provide tools for NMR analysis [10,44] (Figure 4B). Labeled and unlabeled precursor fragments can be produced within a single culture. The use of a dual expression system allows for sequential expression of the precursors in media enriched with different isotopes. Incorporation of unlabeled solubilizing tags into isotopically labeled target proteins was demonstrated by sequential overexpression of unlabeled domain B1 of the immunoglobulin binding protein G (GB1) and labeled prion-inducing domain of yeast Sup35p, each fused to *Ssp* DnaE split intein fragments. PTS resulted in production of the protein fusion with improved stability and solubility created by the NMR-invisible tag [44].

Recently, several intein-based methods for selective protein labeling inside of living cells have been developed. All of these methods employ newly developed split inteins with very small N- or C-intein fragments, ranging from 6 to 15 amino acids [45-49]. Because the intein fragments are so short, they are easier to synthesize and more likely to penetrate the cell to allow for *in vivo* labeling. Intein-based *in vivo* labeling has several advantages over methods based on molecular recognition and chemical modifications [50]. As opposed to direct chemical modifications, intein-based labeling relies on intein-based recognition and minimizes the background from non-reacted reagents.

One such intein-based labeling method uses native chemical ligation, in both bacterial and mammalian systems, to label glutathione-*S*-transferase (GST) and eGFP *in vivo*. The target proteins are expressed as a C-terminal fusion to the *Ssp* DnaB intein, and are designed to have an N-terminal Cys after intein C-terminal cleavage. The target proteins are then labeled by a cell-permeable, thioester-containing small molecule tag such as biotin or a fluorophore [51]. However, this ligation is inefficient because the label and the target have no native affinity for one another, and therefore excess of one reagent needs to be used, resulting in high background signal. Such difficulty was overcome by Camarero and coworkers, who utilized PTS to increase the affinity between target protein and probe, and used a quencher to reduce the signal from unreacted reagent [52] (Figure 4C). The quencher was introduced to the C-terminal *Ssp* or *Npu* DnaE intein fragment, while the fluorophore was part of the C-extein. Therefore, the quencher and fluorophore were part of the same molecule before splicing. On PTS, the fluorophore is ligated to the protein of interest and dissociated from the I_C_-linked quencher.

A PTS-based site-specific conjugation of a quantum dot to the C terminus of pleckstrin homology (PH) domain was performed using the *Ssp* DnaE mini-intein inside of *Xenopus* embryos [53]. The authors speculate that their technique allows for covalent conjugation of any nanostructure and/or nanodevice to any protein within the cells of the developing embryo. Later the same group showed that the approach can be extended to accomplish N-terminal protein tagging using the *Ssp* DnaB mini-intein [54]. This work demonstrated the possibility of site-specific conjugation of quantum dots to several proteins simultaneously, allowing for multi-parameter imaging [54].

### Regulation of protein function by conditional protein splicing

For protein splicing to regulate the activity of a protein *in vivo*, it must splice conditionally, either in *cis* or in *trans.* CPS is activated by a trigger, such as a small molecule, light, temperature, pH or change in redox state (reviewed in [7]). For CPS to be physiologically relevant, it remains to be shown that native inteins are sensitive to stimulus in their native extein contexts, expressed in their host organism. Such evidence would counteract the belief that inteins persist solely as selfish genetic elements, and are difficult to remove because they interrupt key proteins such as DNA polymerase and recombinase [55]. Rather, some inteins still may play a beneficial role for their host, which might provide a positive selective pressure to retain the intein.

#### Small molecule induced CPS

**Figure 5:**
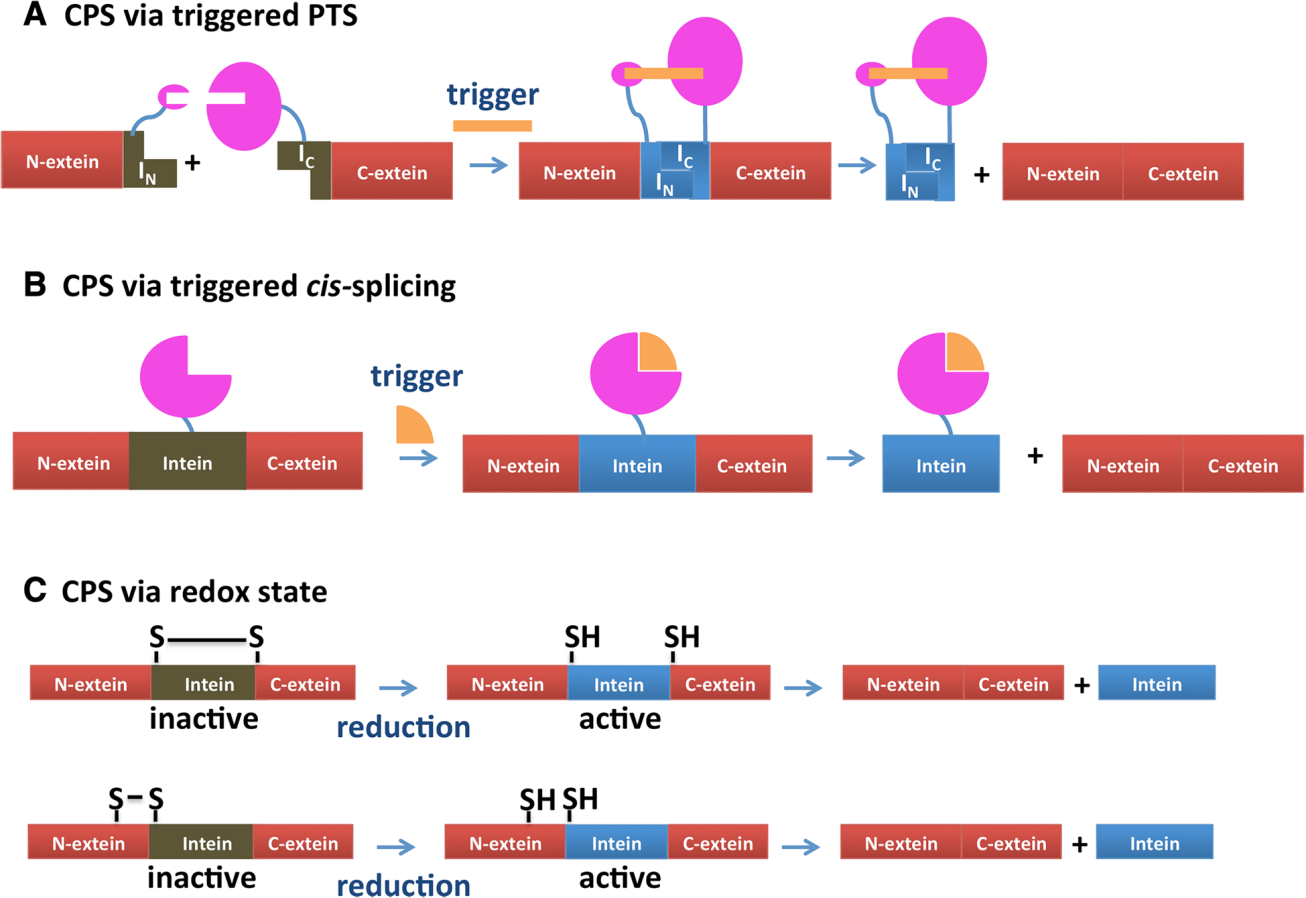
**Schematic representation of conditional protein splicing (CPS).** The brown color of the boxes indicates an inactive intein, whereas a blue intein is active. **A.** Conditional protein splicing triggered by protein *trans*-splicing. **B.** Conditional protein splicing triggered by protein *cis*-splicing. **C.** Conditional protein splicing triggered by change in redox state.

PTS facilitated by ligand-induced dimerization domains allows for activation of splicing by small molecules (Figure 5A). The *Sce* VMA intein was split and fused to rapamycin binding domains FKBP12 and FRB, such that the addition of rapamycin induces intein reassociation and PTS [56,57]. This has found *in vivo* applications such as the controllable generation of firefly luciferase in cultured cells and in *Drosophila melanogaster*[58], and PTS of a tobacco etch virus protease in yeast [59]. A mutated form of FKBP12 can be used to induce spontaneous reassociation and PTS of the split intein; in this case, the addition of rapamycin prevents reassocation and inhibits splicing [60]. Recently, Silver and coworkers demonstrated that the FKBP12 and FRB domains could be replaced with complementary coiled coil domains to induce luciferase activity in mammalian cells via specific coiled coil interactions rather than addition of a small molecule, presumably by inducing PTS of the luciferase segments [61].

Engineered inteins also have been created to control *cis*-protein splicing (Figure 5B). The *Mtu* RecA intein was interrupted by the human estrogen receptor ligand binding domain in place of its endonuclease domain and modified by directed evolution to splice only with the addition of 4-hydroxytamoxifen in *S. cerevisiae*[62]. This was extended to mammalian cells, to facilitate splicing of fluorescent reporter proteins and transcription factors that facilitate the hedgehog pathway [63,64], and to create a CPS-activated histone H2A variant in *S. cerevisiae*[65]. The *Mtu* RecA intein was also interrupted by the human thyroid hormone receptor β and shown to control splicing of β-galactosidase and β-lactamase in *E. coli* in response to thyroid hormone [66].

Therefore, it is possible to design inteins that can be turned either on or off with a small molecule. Such small molecule control of splicing may be used to control the active of native, non-engineered inteins. For instance, cisplatin has recently been discovered to inhibit protein splicing both *in vitro* and in *E. coli* and in *M. tuberculosis*[67] and divalent cations can prevent protein splicing *in vitro*[68-73].

#### CPS activated by temperature, light or pH

Perrimon and coworkers developed a temperature-sensitive version of the *Sce* VMA intein that allows for timed control of protein splicing induced by temperature changes, both in yeast and *D. melanogaster*[74]. The intein introduces control of the activation of the transcription factors Gal4 and Gal80, which in turn allows for temperature-dependent activation or repression of the transcription of target genes. This is exciting as it could allow for more general control of protein activity by CPS, given that control is linked to transcriptional activation, which could theoretically control any gene, rather than linked to interrupting a specific target protein by post-translational control. The temperature range was recently broadened by mutagenesis [75], and was used to control the activity of a T7 RNA polymerase in *E. coli,* and hence to control the expression of *lacZ* under control of the T7 promoter [76]. The temperature sensitive mutant was also used to conditionally activate an essential gene in *Dictyostelium discoideum* to identify the function of the gene that is associated with a disorder that predisposes patients to leukemia [77]. Such temperature-dependent CPS activity might have general physiological relevance, as native inteins from extreme thermophiles have been shown to be conditional on elevated temperature for activity [78-85].

PTS also can be regulated by photoactivation, either by intein fusion to a photodimerization domain [86] or by the addition of protecting groups that are photo-cleavable [87,88], as reviewed in [7]. More recently, Mootz and coworkers have designed a split *Ssp* DnaB intein than can induce C-terminal cleavage on irradiation. They used this system to liberate staphylocoagulase from the I_C_ segment, which in turn activated native prothrombin, both *in vitro* and in plasma [89]. Protein splicing side reactions may also be enhanced by changes in pH [90].

#### CPS induced by reduction

CPS can be controlled by the redox state of a disulfide bond that prevents an intein fusion protein from promoting splicing or side reactions (Figure 5C). For instance, the isolation of an unspliced precursor via *in vitro* PTS can be facilitated by reassociation of split intein fragments under oxidizing conditions, with activity induced by the addition of reducing agents [18,91]. Recently, a study in mice showed that a disulfide bond between N- and C-extein residues improved PTS facilitated by the split *Ssp* DnaE intein, as measured by extein activity [92]. Premature *in vivo* cleavage or splicing of *cis-*splicing inteins can also be controlled by the introduction of Cys residues in intein or extein positions in order to use inteins in biotechnology applications [93] or to study the mechanism of splicing [94].

Lately, there has been evidence that this disulfide-bond control of splicing activity may have physiological relevance. Belfort, Callahan and coworkers designed a redox trap into the fused, *cis-*splicing version of the *Ssp* DnaE intein, by introducing a Cys (Cys-3) residue in the N-extein. This intein could facilitate N-terminal cleavage only under reducing conditions in *E. coli* and allows for purification of uncleaved precursor and subsequent *in vivo* cleavage after addition of reducing agents [95]. This intein redox trap was subsequently used as a FRET-based biosensor for cellular redox state, showing that inteins can control the response of exteins by their conditional activity [96]. They also discovered the MoaA intein from *Pyrococcus abyssi* has a native disulfide bond also between Cys-3 and Cys1 that can control intein activity [95]. The *Pab* PolII intein has a disulfide bond between Cys1 and Cys + 1 that prevents splicing, and the influence of flanking extein residues on both splicing activity and disulfide bond formation appears to be linked [97]. The activity of the *Mma* PolII intein is dependent on an internal intein disulfide bond, and shows differential splicing activity based on the redox state of the *E. coli* strain or localization to the periplasm or cytoplasm [98].

### Intein-based biosensors

**Figure 6:**
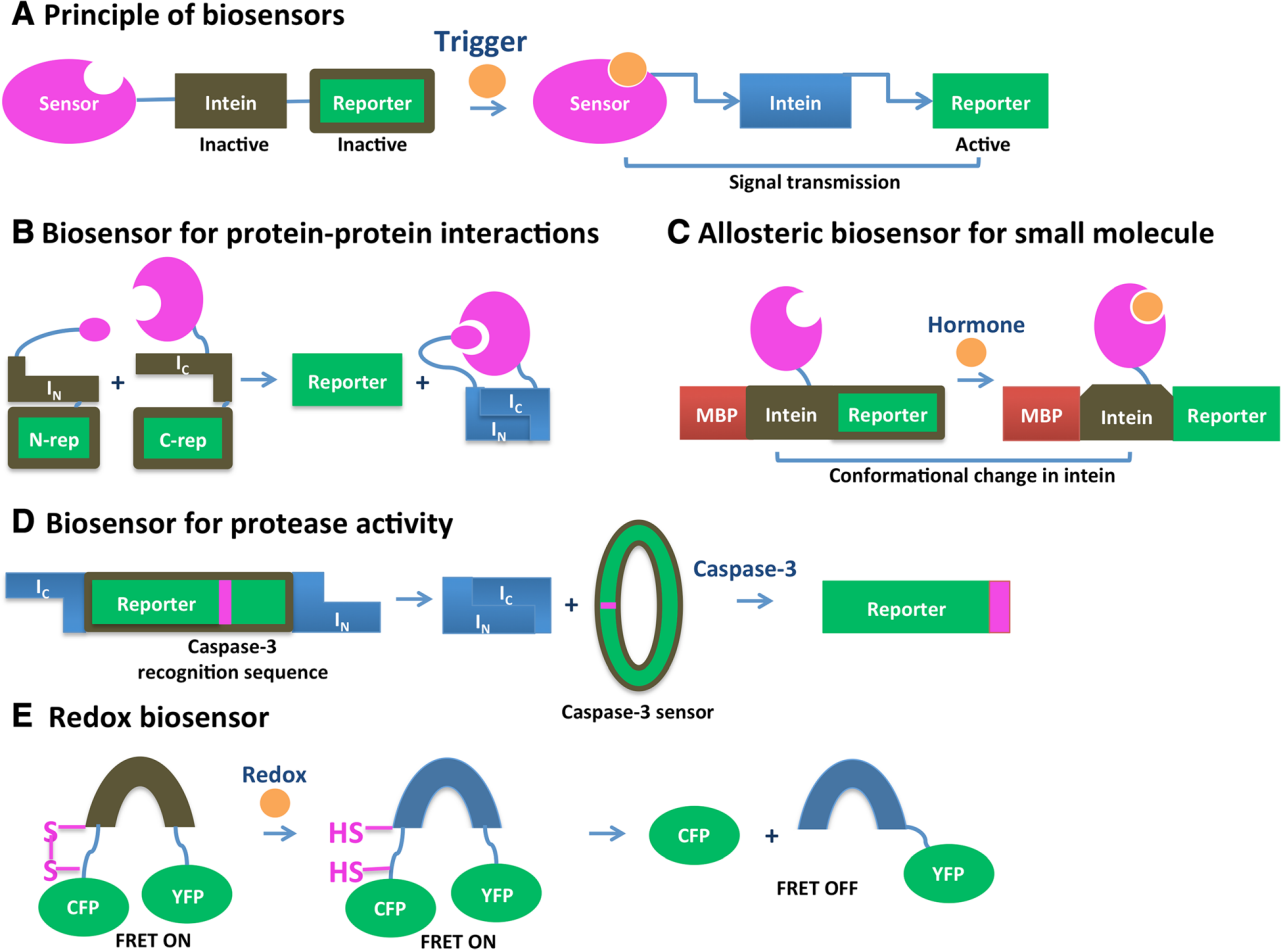
**Schematic representation of intein-mediated biosensors.** Segments either colored brown or enclosed in a brown box indicate an inactive intein or reporter. **A.** Overview of use of an intein as a biosensor. **B.** Use of an intein-based system to sense protein-protein interactions. **C.** Intein-mediated allosteric biosensor for small molecule. **D.** Intein-mediated biosensor for protease activity. **E.** Intein-mediated redox biosensor. CFP and YFP are cyan and yellow fluorescent proteins, respectively.

CPS permits splicing in response to a specific trigger and lays a foundation for the development of intein-based sensors. Most of these sensors have three functionally and structurally distinct modules: a sensing module, an output module and intein-derived signal transducer (Figure 6A). Signal recognition by a sensor module leads to CPS or conformational changes of the intein connector and activation of the reporter protein. An advantage of intein-based sensors is that the modular design allows for easy exchange of sensor and reporter elements, and, in cases where splicing is involved, the presence of the intein may be trace-less after sensing. Intein-based biosensors have been developed to detect protein-protein interactions, changes in DNA methylation patterns, protein trafficking, small molecules, protease activity and redox state of the cell.

#### Sensing protein-protein interactions

Intein biosensors for protein-protein interactions utilize PTS facilitated by split intein fragments that have low binding affinity for each other. Design of these biosensors involves creation of two fusion proteins, each containing one protein binding partner, a split intein fragment and a fragment of a reporter protein (Figure 6B). Interaction of the binding partners facilitates split intein reconstitution and splicing-induced complementation and activation of a reporter protein. Umezawa and coworkers applied this sensor design to demonstrate protein-protein interactions in various *in vivo* systems ranging from *E. coli* to transgenic animals. In their original work, an *E. coli*-based biosensor was developed to monitor binding between calmodulin and its target peptide M13, using GFP reconstitution as the reporter, mediated by the artificially split *Sce* VMAI intein [99]. Next, an insulin-induced interaction between phosphorylated insulin receptor substrate 1 and its target (the N-terminal SH2 domain of PI 3-kinase) was observed in mammalian cells by luciferase reconstitution by the naturally split *Ssp* DnaE intein [100]. Then, they demonstrated a bioluminescence imaging method to noninvasively and quantitatively image protein-protein interactions in mice by intein-mediated reconstitution of split firefly luciferase proteins driven by the interaction of two strongly interacting proteins, MyoD and Id [101]. To increase the sensitivity of detection, protein splicing was employed to produce a functional transcription factor that modulates a reporter gene, firefly luciferase [102,103]. In this work, the epidermal growth factor (EGF)-induced interactions of an oncogenic product Ras and its target Raf-1 was monitored by bioluminescence signals in mammalian cells. Notably, this interaction was not detected by traditional two-hybrid systems.

#### Sensing DNA methylation

Similar biosensor design was utilized in living cells for reporting sequence specific changes in DNA methylation via luminescence [104]. The biosensor design comprises two fusions, each with a polydactyl zinc finger domain fused to a split intein fragment and to a split-luciferase domain. The luciferase reporter can be reconstituted by conditional protein splicing upon binding of two polydactyl zinc finger domains to their DNA targets. This biosensor is capable of detecting the loss of epigenetic silencing and increased accessibility of a DNA sequence near the promoter region of the L1PA2 subfamily of Line-1 retro-elements upon treatment with a demethylating drug.

#### Sensing protein localization and internalization

Intein biosensors can be used to perform high-throughput screens to identify protein localization in a specific compartment. Design of these biosensors involves creation of two fusion proteins, each containing a split *Ssp* DnaE intein fragment and a fragment of a reporter protein (GFP or luciferase). In addition, one of the fusion proteins has a target protein and the other is designed to localize in a specific compartment, such that PTS can only occur in the appropriate cellular compartment. This sensor design was used to study translocation of protein to the nucleus [105] or mitochondria [106], as well as release of proteins from the mitochondria to the cytosol [107].

In addition to the detection of protein subcellular localization, protein splicing has been used for quantitative analysis of G-protein-coupled receptor (GPCR) internalization. In this case, interaction between activated GPCR and intracellular beta-arrestin2 results in *Npu* DnaE intein-mediated reconstitution of luciferase [108].

A protein localization-dependent sensor also was developed for corticosterone detection in animals [109]. Again, the biosensor has two components. The first is a cytosol-localized fusion of glucocorticoid receptor with C-terminal fragments of the *Ssp* DnaE intein and split luciferase. The second is a nucleus-localized fusion of the N-terminal fragments of the intein and luciferase. Upon corticosterone binding, the glucocorticoid receptor is translocated into the nucleus, facilitating intein fragment complementation and splicing and therefore activation of luciferase.

#### Sensing small molecules

Allosteric intein biosensors can be used to detect small molecules. Wood and coworkers have designed sensors for ligand detection by human nuclear hormone receptors utilizing allosteric effects induced by ligand-receptor binding. The sensor does not rely on protein splicing; the intein is used as an allosteric transmitter that allows communication between the hormone receptor and the reporter. The sensors exploit a four-domain fusion protein in which a nuclear receptor of interest is inserted in a loop region of the non-splicing *Mtu* RecA intein. The intein is fused to the *E. coli* maltose binding protein and a T4 bacteriophage thymidylate synthase reporter (Figure 6C). The biosensor is based on a thymidylate-synthase deficient *E. coli* cell strain expressing the fusion protein, such that cell growth depends on the thymidylate synthase reporter activity modulated by presence of the hormone in a dose-dependent manner. Initially, the human estrogen (ERα) and thyroid hormone (TRβ-1) receptors were used to develop highly sensitive methods for detection of nuclear hormone receptors ligands [66]. Later, an optimized estrogen sensor was created that is capable of identifying diverse estrogenic compounds and differentiating between their agonistic/antagonistic pharmacological effects [110]. Subtype-specific nuclear hormone receptor sensors were developed for estrogen [111] and thyroid hormone [112] receptors using the human estrogen (ERα and ERβ) and the thyroid (TRα-1 and TRβ-1) receptors as sensing domains. The peroxisome proliferator-activated receptor gamma (PPARγ) ligand binding domain was used to create a series of bacterial biosensors for the identification of functional PPARγ ligands [113]. This study showed that the linker region between the intein and the thymidylate synthase reporter influences the quality of the transmission of the allosteric signal induced by ligand binding.

A splicing-dependent allosteric intein biosensor was employed by Liu and coworkers for construction of an *E. coli*-based estrogen detector [114]. The sensing element of this system is the estrogen-sensitive *Sce* VMA(ER) intein that was generated by replacement of the endonuclease region with the human estrogen receptor α. The VMA(ER) gene was inserted in the constitutively expressed chromosomal *lacZ* gene. The principal difference of this sensor from that discussed above is that the detection here relies on estrogen-dependent intein splicing and activation of the reporter protein.

#### Sensing protease activity

A biosensor for protease activity was developed based on *in vivo* intein-promoted protein cyclization [29] (Figure 6D). Firefly luciferase was fused to a caspase-3 recognition sequence and cyclized by the inverted *Ssp* DnaE split intein. In the absence of caspase activity, the activity of the cyclized luciferase was diminished due to steric hinderance. However, the activity of luciferase is fully restored upon caspase-dependent cleavage, enabling real-time quantitative sensing of caspase-3 activity in mice.

#### Sensing oxidation state

A bacterial redox sensor was developed utilizing the disulfide-bond control of the *Ssp* DnaE intein splicing activity [96] (Figure 6E). The *Ssp* DnaE intein with an engineered disulfide trap is inactive in the oxidized form and triggered by a reducing environment to produce N-terminal cleavage [95], as described above. This redox sensitive intein was fused with a FRET reporter to detect hyperoxic *E. coli* mutants.

### Delivery and control of *trans-*genes

#### *Delivery and control of* trans*-genes* in plantae

Inteins have been used to control *trans*-gene expression; the initial examples were in plants and were reviewed by Evans and coworkers in 2005 [115]. Briefly, plant genes were first split and fused with segments of split inteins, with extein activity demonstrated in *E. coli.* The advantage of transferring genes that confer desired traits as split genes is that it minimizes the chance of transfer of the gene to unwanted hosts, such as transfer of herbicide resistance from crops to weeds, since the weed would need to receive both fragments of the gene separately. For example, the split site for acetolactate synthase (ALS) was determined by rational design, and *trans-*splicing to produce herbicide-resistant ALS was demonstrated in *E. coli*[116]. The split site for *Salmonella typhimurium* 5-enolpyruvylshikimate-3-phosphate synthase (EPSPS) was selected by a library-based approach in *E. coli,* and the split intein segments facilitated EPSPS activity via fragment reassociation, to create an active split EPSPS protein, with PTS not required [117,118]. This subtlety is important to note when evaluating claims that PTS is responsible for trans-gene activity *in vivo*. Full-length EPSPS was later generated by PTS, with expression directed to the chloroplast, in *Nicotiana tabacum*[118]. This was reproduced with a more highly herbicide resistant EPSPS from *Pseudomonas fluorescens* both in *E. coli* and in *N. tabacum*[119].

A split intein was also used to generate functional, transgenic β-glucuronidase (GUS) in *Arabidopsis thaliana*, both by PTS and by intein-mediated reassociation of GUS fragments [120]. The split GUS-intein fusion studies were extended to demonstrate that PTS occurs via plasmid-induced expression in leaf cells of soybean, pea, maize and barley [121].

Functional reconstitution of the barnase from *Bacillus amyloliquifaciens* can be facilitated by the split version of the *Ssp* DnaB intein [122]*.* The split genes were placed under the control of a promoter for the tapetum, such that reconstitution of the barnase results in male sterile plants, given that the ribonuclease activity of the barnase is toxic to the tapetum cells in the anther [123]. The split barnase can be activated by split intein segments to facilitate cytoxicity via transient agroinfiltration of leaves from *N. benthamiana* or to result in male sterility in transgenic *A. thaliana*. Rather than select for the split barnase genes with separate genetic markers, each fragment was genetically linked to a split gene for an acetolactate synthase (ALS)-intein fragment fusion [19]. Therefore, expression of both the resistance gene for sulfonylurea herbicides (ALS) and expression of the toxic barnase require reconstitution of separate split intein pairs [116]. Although PTS was not definitively shown by a biochemical assay, the split genes did function as predicted to allow for selection of male sterile *A. thaliana*[123]. This work has been extended to demonstrate PTS in transgenic *Triticum aestivum*[124,125].

Intein-mediated and thermoregulated control of transgenic maize has been recently described with a split xylanase, which when active breaks down plant cell walls [126]. Building on their computational analysis of the characteristics of intein insertion sites [127], Raab and coworkers interrupted a thermostable xylanase from *Dictyoglomus thermophilum* with the DnaE-1 intein from *Thermus thermophilus*[126]. Random mutagenesis via error-prone PCR was used to generate mutants of the xylanase-intein fusion protein that are splicing active only at high temperature but retain wild-type xylanase activity. Transgenic maize expressing uninterrupted and active xylanase produces shriveled seeds with low seed mass, but maize expressing the xylanase-intein fusion produces normal seeds, suggesting that toxic xylanase activity is prevented by the intein insertion. The maize that expresses either xylanase produces more glucose during processing. Therefore, the thermoactive intein allows for the xylanase to be expressed but not active during maize growth, when it would be toxic, and then to be active during bio-mass processing, when it is useful.

A split DnaB intein was used to facilitate polymerization of spider silk flagelliform protein in tobacco plant leaves by both stable and transient transfection [32] (Figure 3C).

#### Trans-*genes in other organisms*

Recently, PTS in mammalian cells and in mice has been used to test delivery of *trans-*genes by adenovirus delivery vectors. The split fragments of the *Ssp* DnaE intein were fused to heavy and light chain genes for the B-domain deleted factor VIII, and delivered to mammalian cells or mice by separate viral vectors [92,128]. Splicing activity is suggested by increased coagulation activity and the concentrations of functional protein in the plasma, suggesting that PTS could be used for the *in vivo* generation of proteins too large to be delivered by traditional viral vectors. The split *Ssp* DnaE intein also was used to facilitate split Cre reconstitution in mice. In short, fragments of split Cre recombinase can be fused to separate promoters that drive expression under different conditions. Under conditions where both Cre fragments are expressed, Cre is reconstituted and facilitates expression of genes under control of the Cre-LoxP system. The *Ssp* DnaE intein was shown to help improve functional Cre fragment complementation [129].

## Conclusions

It is exciting to observe that biotechnology applications of protein splicing have begun to move from proof-of-concept experiments to productive applications in which the intein is the tool rather than the object of study itself. However, the variability in how inteins behave in heterologous contexts can be a limiting factor in the general applicability of intein-based biotechnology. Going forward, recent advances in the understanding of the role of flanking extein residues in the splicing process [130-136] may improve our ability to predict or ameliorate this challenge. The discovery of split inteins that splice even faster than the *Npu* DnaE intein [137] also may increase the efficiency of PTS-based applications. The growing number of examples of *in vivo* protein manipulation using intein catalysis also promises advancement in intein-based tools for systems and functional biology.

## Abbreviations

ACP: acyl carrier protein; ALS: acetolactate synthase; CHO: Chinese hamster ovary; CPS: conditional protein splicing; eGFP: enhanced GFP; EPL: expressed protein ligation; EPSPS: 5-enolpyruvylshikimate-3-phosphate synthase; ER: human estrogen receptor; GB1: domain B1 of the immunoglobulin binding protein G; GFP: green fluorescent protein; GPCR: G-protein-coupled receptor; GST: glutathione-*S*-transferase; GUS: β-glucuronidase; HC: heavy chain; Hay: *Haemophilus parainfluenzae*; IC: C-terminal intein fragment; IN: N-terminal intein fragment; IP: intein-derived protease; LC: light chain; mRFP: the monomeric red fluorescent protein; MHC: major histocompatibility complex; Mtu: *Mycobacterium tuberculosis*; Pch: *Penicillium chrysogenum*; PH: plextrin homology; POI: protein of interest; PTS: protein *trans-*splicing; Sce: *Saccharomyces cerevisiae*; Ssp: *Synechocystis* sp. PCC6803; TR-1: human thyroid receptor

## Competing interests

The authors declare that they have no competing interests.

## Authors’ contributions

NT and KM co-authored the article and read and approved the final manuscript.

## Authors’ information

NT is a post-doctoral scholar in the lab of Professor Marlene Belfort at the University at Albany; KM is associate professor and chair of the chemistry department at the College of the Holy Cross.
